# Global, highly specific and fast filtering of alignment seeds

**DOI:** 10.1186/s12859-022-04745-4

**Published:** 2022-06-10

**Authors:** Matthis Ebel, Giovanna Migliorelli, Mario Stanke

**Affiliations:** 1 Institute for Mathematics and Computer Science, University of Greifswald, Walther-Rathenau-Str. 47, 17489 Greifswald, Germany; 2 Center for Functional Genomics of Microbes, University of Greifswald, Felix-Hausdorff-Str. 8, 17489 Greifswald, Germany

**Keywords:** Spaced seeds, Alignment, Alignment anchor, Genome alignment, Geometric hashing

## Abstract

**Background:**

An important initial phase of arguably most homology search and alignment methods such as required for genome alignments is *seed finding*. The seed finding step is crucial to curb the runtime as potential alignments are restricted to and *anchored at* the sequence position pairs that constitute the seed. To identify seeds, it is good practice to use sets of spaced seed patterns, a method that *locally* compares two sequences and requires exact matches at certain positions only.

**Results:**

We introduce a new method for filtering alignment seeds that we call *geometric hashing*. Geometric hashing achieves a high specificity by combining non-local information from different seeds using a simple hash function that only requires a constant and small amount of additional time per spaced seed. Geometric hashing was tested on the task of finding homologous positions in the coding regions of human and mouse genome sequences. Thereby, the number of false positives was decreased about million-fold over sets of spaced seeds while maintaining a very high sensitivity.

**Conclusions:**

An additional geometric hashing filtering phase could improve the run-time, accuracy or both of programs for various homology-search-and-align tasks.

**Supplementary Information:**

The online version contains supplementary material available at 10.1186/s12859-022-04745-4.

## Background

Aligning two or more genomic (or protein) sequences is one of the most fundamental tasks in bioinformatics. A base assumption is that if two sequences align well, they are likely to share a common evolutionary origin, i.e. are homologs. Often, one is especially interested in finding *orthologies* which might indicate the same function. The alignment of whole-genomes is instrumental to comparative genomics and comparative genome annotation in particular [1]. The number of newly sequenced genomes can be expected to continue to grow for a long time. For example, the Vertebrate Genomes Project aims to generate reference genome assemblies of about 70,000 vertebrate species [2]. Creating a whole-genome (multiple) alignment requires to construct many local alignments of evolutionary related fragments of the different genomes. The task to find homologous genomic regions is of increasing importance and accurate, efficient and scalable methods are needed [1]. Thereby, most truly homologous, nonrepetitive genomic regions (e.g. coding exons of orthologous genes) shall be found but the number of hits of unrelated or repetitive regions shall be limited.

A common approach to alignment is the seed and extend approach [3]. In a first step, very short local similarities are sought. For the sake of speed, these similarities may be required to be identities. The resulting hits constituting of one *fragment* per sequence then serve as *alignment anchors*. In a second step, starting from these anchors, local alignments are computed. As the second step is usually more time consuming, it is important that the anchors, also called *seeds*, from the first step are sensitive and specific, while being found very quickly. As sensitivity and specificity can be traded off against each other we will sometimes generally refer to accuracy. This work focusses on finding these seeds and improving established methods to do so.

In early aligners, small exact matches of length *k*, so called *k*-mers, were used as seeds [3]. Ma et al. [4] and Burkhardt and Kärkkäinen [5] were among the first to introduce the idea of *spaced seeds*, where bases at certain positions of a seed—so called *don’t care* positions—are not required to match. They independently found that the positions, at which mismatches are allowed, have a high influence on the sensitivity of spaced seed patterns. In a following article, Li et al. [6] introduced multiple spaced seeds and use a *set of spaced seed patterns* to find alignment anchors with even higher sensitivity. Much research has been carried out investigating the optimality of spaced seed patterns and the hardness to actually compute such patterns (e.g. [7–20], among others), usually under the assumption of very simple probabilistic models of homologous and non-homologous sequence pairs. There are special types of spaced seeds, such as transition-constrained seeds as used in BLASTZ [21] and YASS [22] (among others), that allow transitions at certain seed positions (i.e. allow an A-G or a T-C match). Extending spaced seeds this way can further improve seed accuracy [22, 23] and is a compatible replacement to the plain spaced seeds used in our work. The interested reader is referred to this survey by Brown [24] for a more detailed overview of the topic.

Spaced seeds have been applied in alignment software such as DIAMOND [25], LASTZ [26], YASS [27] and the discontiguous MegaBLAST version of BLASTn [3, 28, 29], to name a few. In previous research on further improving spaced seeds, Noé and Kucherov [22] introduced an additional filter criterion. A local group of possibly overlapping individual seeds is treated as a unit when they are proximate to each other and on close diagonals. Then the total number of matching bases of such a seed group is thresholded to trigger an alignment in YASS. The idea of considering the number of seeds in a diagonal “band” or “bin” as a filter criterion was also previously described by e.g. Rasmussen et al. [30] and Meyers [31]. Mak et al. [32] presented “indel seeds” that can cope with very small indels in homologous regions, thereby sacrificing speed. Recently, Leimeister et al. [33] applied spaced seeds with an additional filtering step in a multiple sequence alignment pipeline. They used very sparse spaced seed patterns with 10 match and 100 don’t care positions and a novel filtering step, scoring *all* positions in a seed to filter out noise.

Seed-*chaining* can also be used as an explicit filter on seeds. For example, Abouelhoda et al. [34] published the CoCoNUT seed finding and alignment software tools, which use maximum unique matches as seeds. As a phase during alignment, subsets of seeds whose fragments are in the same order in all sequences (*chains*) are selected. Such a chaining phase would be a natural next step after our filtering approach.

In this article, we compare fast methods to increase sensitivity and specificity of (spaced) seeds. We here consider methods that are based on exact re-occurrences of fixed-length sequence patterns, which can be implemented very efficiently and thus can be used as a very fast initial step in an alignment pipeline. Typically, such seeds could be in a particularly well-conserved region with higher sequence similarity and would be used as starting point for a sequence of steps that constructs a local alignment that extends further and uses a detailed scoring to increase the specificity of the hit. Subsequent filtering steps can be slower than the seed finding and typically include a gapless extension of anchors (e.g. with the X_drop heuristic [3]), thresholding the alignment score of the extension, the joining of nearby gap free alignments and local alignment extensions that may contain gaps and further thresholding ([3, 26]). The seeds can be interpreted as *anchors* that constrain the set of admissible alignments, significantly reduce their number and also the runtime of alignment algorithms.

We introduce a novel method, *geometric hashing*. Geometric hashing is a fast filter of candidate seeds taken, such as exact *k*-mer matches induced by spaced seed patterns. To achieve a higher accuracy, the matches from homologous regions are accumulated over possibly *long distances* using a secondary hashing technique. We evaluate the methods on real genomic data from human and mouse for sensitivity, as well as on artificial random sequences to assess specificity. Geometric hashing can be adjusted to simultaneously be more sensitive and much more specific than existing methods at finding seeds in coding regions of homologous genome regions and requires only a small fraction of additional runtime.

We confirm that multiple spaced seed patterns are better than a single spaced seed pattern. On this task, sets of four spaced seeds produce one to two orders of magnitude fewer false hits than a single spaced seed pattern. We also confirm and quantify the superiority of spaced seeds over contiguous *k*-mers as seeds in finding homologous exons.

*Geometric hashing* can be adjusted to decrease the number of false positives by at least six orders of magnitudes while maintaining the sensitivity. Alternatively, when using a *k* that is one smaller than the *k* used by a set of four spaced seed patterns, here $k=14$ versus $k=15$, geometric hashing simultaneously reduces the false negatives by 19.5% from 4.6% to 3.5% and the number of false positives by a factor of about $2\cdot 10^5$.

## Methods

### Test data

As test data set we used genomic sequences from the softmasked genome assemblies of human and mouse:

- *Homo sapiens* (hg38 GCA_000001405) [35]
- *Mus musculus* (mm10 GCA_000001635) [36]

Ortholog protein coding genes have been queried from Biomart [37], the Ensembl interface to access homology predictions of genes. Pairs of ortholog genes were selected, thereby ensuring a high confidence in the orthology relation according to their respective Ensembl score [38]. Further, only one-to-one orthologs were allowed in the dataset, such that each sequence appears in only one pair and no two sequences from different pairs were considered orthologs.

We retrieved the GFF genome coordinate files containing Ensembl annotations for the human genome, a single representative principal transcript was picked for each gene in order to avoid any bias towards those genes which include a larger number of transcripts. We added flanking regions to both ends of each gene. The lengths of these flanking regions were randomly and independently chosen from [5000, 10000]. This was done to prevent a potential bias from assuming relative gene boundaries.

We selected a subset of 705 pairs of human and mouse gene regions, each containing one orthologous gene pair. The average sequence lengths were 88 kbp and 80 kbp for human and mouse, respectively. The total length of human and mouse genic regions in the test set were approximately 62 Mbp and 56 Mbp, respectively.

As real genome sequences cannot be guaranteed to be void of further homologies besides the chosen orthologies, we simulated a set of random sequences for an estimation of the number of false positives. For each real sequence in the human-mouse dataset, we simulated a random DNA sequence with independent and uniformly distributed nucleotides of the same length as the respective gene, labeling it with the respective genome. Choosing this simple distribution for the negative examples is in agreement with most previous work on spaced seeds. The independent and uniform distribution is stated either explicitly [24, 39] or is implied by considering all seeds of length or weight *k* as equally specific [11, 40]. All seed hits in this artificial set of DNA sequences were counted as false positives.

### Evaluation

As spaced seeds have generally been designed to anchor an alignment of *two* sequences, we will evaluate and compare all methods on a *pair* of genomes as well, here from human and mouse. However, the geometric hashing idea generalizes to more than two genomes. Formally, we define an alignment seed as a quadruple $(S_1, i, S_2, j)$, where *i* is a position in sequence $S_1$ and *j* is a position in sequence $S_2$. The seed can be interpreted as a prediction that these two positions are believed to be homologous positions. The applied methods actually rather identify small region pairs of equal length, e.g. of length *k*. We therefore use for evaluation purposes the region midpoints.

Several alignment anchors could eventually lead to the same local alignment of homologous regions. This is to be expected for seeds $(S_1, i, S_2, j)$ and $(S_1, i', S_2, j')$ of the same sequence pair, where $i'-i$ and $j'-j$ are small and similar or even equal. It is therefore sufficient to find at least one of the anchors that are redundant in this sense, which motivates the following accuracy measure.

#### Sensitivity

We say a seed $(S_1, i, S_2, j)$ *supports* a coding sequence (CDS) with coordinate range [*a*, *b*] of sequence $S_1$ if $a\le i\le b$ and if $(S_1,S_2)$ is a pair of homologous gene regions. We calculate the percentage of human coding exons (CDS) with *at least one* supporting seed and define

$$\begin{aligned} \text {sensitivity} := \frac{\text {number of supported human CDS}}{\text {number of human CDS}} \end{aligned}$$

This measure is based on the notion that the alignments of coding sequences of homologous CDS pairs typically contain only small numbers of indels, are relatively highly conserved and therefore a subsequent alignment anchored in such a seed would likely result in an alignment of at least a large part of the exons. Brejova, Brown and Vinar find on human-mouse homologous coding gene pairs that the CDS alignment fragments, that are not interrupted by introns, are on average 152 bp long. This figure reduces to 120 bp if the fragments are considered to end at an indel [10].

Note that we did not require that the corresponding position *j* in mouse is homolog. One reason for this choice is that the accuracy measure would otherwise depend on the completeness and correctness of some reference alignment and would also presuppose that a matching splice form is annotated and identified. The sensitivity would then have an unknown upper limit $<1$ that depends on other tools and their settings. Another reason is that we will below consider limits to the overall number of false positives such that the number of false positive seeds in homologous region pairs $(S_1,S_2)$, that contain only a single gene each, is negligible. Thirdly, all methods are compared with the same accuracy measures and we do not expect the relative performances to be affected.

Note that this sensitivity measure still cannot quite be expected to achieve 100% because not all human exons have a homologous region in the mouse genome. In order to raise upper limit to the practically achievable sensitivity to 100%, we ignore a small number of human exons which we expect cannot be anchored, e.g. because the gene sequences at the respective annotated coordinates are masked or there is no annotated alignment for the exon inside the ortholog mouse sequences [41]. Nevertheless, we consider choices of *k* and other parameters most relevant when the sensitivity is at least 0.9.

#### False positives

To measure and compare the prediction of wrong seeds we applied all approaches also to the set of random sequences described in “Test data” section. Any hit between a random ’human’ and ’mouse’ sequence is considered a false positive (FP). We normalize the number of false positives #FP to $\widehat{\text {FP}}$ as follows

$$\begin{aligned} \widehat{\text {FP}}:= \#\text {FP} \cdot \frac{N\cdot N}{n_{1}\cdot n_{2}} \cdot \frac{1}{N} =\,\frac{\#\text {FP}}{n_{1}n_{2}}N. \end{aligned}$$

#FP is the total number of counted false positives. $n_1\,=\,62.348\cdot 10^6$ and $n_2\,=\,56.197\cdot 10^6$ are the total lengths of the random ’human’ and ’mouse’ sequences from the artificial data set. $N=\,3.22\cdot 10^9$ is the size of the human genome. $\widehat{\text {FP}}$ can be interpreted as the extrapolated fraction of false positive seeds per genome position if two human-sized genomes were compared.

The rationale behind this measure is as follows. The alignment space—more particular, the set of all admissible $(S_1, i, S_2, j)$—is inherently of quadratic size $N^2$ for two genomes of total size *N*. However, through appropriately large *k*’s or thresholds, seed-finding can reduce the number of hits that are further examined to something that is linear in the genome size(s) *N*. An effort that is linear in *N* is unavoidable and acceptable.

### Seeding approaches

In this section five methods M1–5 for seed finding are described in order of increasing accuracy and sophistication. M1 searches for contiguous matches, M2 and M3 use a single and multiple spaced seed patterns, respectively. Methods M4 and M5 use seed candidates found by M3 and apply additional filtering steps to reduce $\widehat{\text {FP}}$.

Matches found by M1-M3 are small similar region pairs, thus we extract the respective midpoints as seed candidates. Repeats in the sequences could lead to many seed candidates originating from the same *k*-mers. Firstly, we do not consider sequence positions that fall in a repeat-masked part of the genome. Secondly, we apply a simple filter to reduce this noise for all methods. If a *k*-mer leads to more than ten seed candidates, we only compute a random subsample of size 10 of all possible seed candidates. The filter value (10) can be adjusted to be more or less strict. Ignoring seeds of patterns with many matches is in accordance with filtering techniques used in other genome aligners, e.g. DIAMOND [25].

For a string *S* and sequence positions $$a\le b$$ let $$S[a..b]$$ denote the substring of *S* from position *a* up to and including position *b*. Exclusion of the end position is denoted with round parentheses, e.g. $$S[a..b)$$ goes up to position $$b-1$$ only. In the following, all sequence positions are implicitly assumed to be in the range of the sequence length. Seed matches with ambiguous or unknown characters (e.g., n) were not considered a match and were discarded.

#### M1: exact contiguous matching

This simplest method serves as a baseline. For a given weight *k*, we say that two sequences $S_1$ and $S_2$ have an exact contiguous match at position pair (*a*, *b*) if and only if

$$\begin{aligned} S_1[a\,..\,a+k) = S_2[b\,..\,b+k). \end{aligned}$$

We then take $(S_1, a+\lceil k/2\rceil , S_2, b+\lceil k/2\rceil )$ as the *seed*, i.e. the center from the two identical substrings of length *k*.

#### M2: spaced seeds

A spaced seed pattern is a binary pattern $p=(p_1,\ldots , p_\ell )\in \{0,1\}^\ell$ of length $\ell$, i.e. a string over the alphabet $\{0,1\}$ where the 1’s are called *match* positions and the 0’s are *don’t care* or *wildcard* positions. The length $\ell$ is called *span* and the number of match positions is its *weight* *k*, with $k \le \ell$. The *k*-mer $x_i$ is the string induced by applying *p* to a sequence *S* at some position *i*, concatenating only the characters from *S* that pair with a match position. More formally, let

$$\begin{aligned} \pi _s := r \text {, such that } p_1+\cdots +p_r = s \qquad \text { for } s = 1..k \end{aligned}$$

be the position of the *s*-th 1 in *p*. The *k*-mer $x_i$ defined by

$$\begin{aligned} x_i[s] := S[i+\pi _s-1] \qquad (s = 1..k) \end{aligned}$$

is said to be the *contiguous pattern induced* by spaced seed pattern *p* at position *i* in *S*.

Let $x_i$ and $y_j$ be the *k*-mers induced by a spaced seed pattern *p* of weight *k* at positions $i-\lceil \ell /2\rceil -1$ in $S_1$ and $j-\lceil \ell /2\rceil -1$ in $S_2$, respectively. We then say that $S_1$ and $S_2$ have a match according to the spaced seed pattern *p* at position pair (*i*, *j*) iff $x_i=y_j$. Note that in the literature about spaced seeds, the notion is often used to refer to the binary spaced seed *pattern*. In this article, we call a seed a pair of sequence positions ($S_1,i,S_2,j$) that could serve as an alignment anchor and write “spaced seed *pattern*” when we refer to the binary pattern of *match* and *don’t care* positions.

It is well-established that the choice of *p* has a significant influence on the sensitivity even at a fixed weight [4, 9]. The sensitivity of spaced seed patterns is related to the number of overlapping hits [42]. Hits of contiguous seed patterns (M1) tend to cluster, while hits of seed patterns with low self-overlap are more evenly distributed and thus more sensitive [11]. Thus, one needs to use optimized spaced seed patterns to get the best results. We used the software SpEED [17, 40] to compute spaced seed patterns of desired weight, that are good under its simplifing assumptions on the distribution of homologous sequences. The underlying model requires the length of the homologous region and the similarity of the homologous region for which the seeds should be optimal. As in [4] we set the region length to 64. The base pair match probability was set to 0.85, the percent identity of corresponding CDS in human and mouse [43].

#### M3: set of spaced seed patterns

Let *P* be a set of $m>1$ spaced seeds patterns, each of weight *k*. We say that $S_1$ and $S_2$ have a match according to *P* at position pair ($i, j$) if the two sequences have a match at this position pair for *any spaced seed pattern* $p\in P$. Li et al. introduced this concept to increase the sensitivity of spaced seed patterns. While reducing the weight *k* of a single spaced seed pattern also leads to higher sensitivity, the trade-off with getting more random hits at the same time is better when using more spaced seed patterns instead [4]. We again used SpEED to generate good sets of spaced seed patterns of desired weight with the same parameters as above.

These first three methods are well known and used as basis and baseline for the upcoming methods. The following methods describe additional filtering steps which can be applied to sets of seeds found by either of the former methods.

#### M4: neighbouring matches

After all matches have been identified, locally the number of consistent matches are counted and a hit is only reported if the number of neighbouring matches reaches a threshold $\tau >1$. The idea is to allow the individual seeds to be less specific (smaller weight *k* or higher number of patterns *m*). Applying this filter, an isolated match that could be random and non-homologous does not necessarily result in a false hit. This method is similar to the one described by Noé and Kucherov [27].

What is considered local versus non-local is controlled by a variable *D*. Suppose $$(S_1,i,S_2,j)$$ is a *candidate* seed from method M3. This position pair is reported as hit *only* if the total number of seeds of some positions ($$S_1,i')$$ and $$(S_2,j')$$, such that $$i'-j' = i-j$$ and $$|i-i'| \le D/2$$, is at least $$\tau$$. As customary, we call the sets $$\{(i,j)\,|\,i-j = \text {const}\}$$ *diagonals* in the pairwise alignment space. In other words, all matches are counted in the region pair of length *D* centered around $$i$$ and $$j$$ that are on the same diagonal. The idea of counting hits on the same diagonal goes as far back as 1983 [44]. Summarizing nearby hits on the same diagonal can be done very efficiently (see “Geometric hashing algorithm” section). As reporting hits on the same diagonal that are very close to each other are likely to be redundant, we do not allow two matches $$(i, j)$$ and $$(i',j')$$ to overlap, i.e. $$|i-i'| \ge \ell$$.

#### M5: geometric hashing

Methods M1–M3 only consider the directly matching regions. Method M4 considers the immediate neighborhood only. In contrast, M5 is able to gather evidence for homology from matches that are distant to each other. The idea is to collect seed candidates from multiple exons from the same orthologous genes. Or, more generally, from seed candidates with similar distance differences in the two sequences. Reporting only seeds from sufficiently large such collections then increases specificity as single random matches typically remain unreported.

Our geometric hashing approach is motivated by an eponymous technique from object recognition in computer vision [45]. There, first distinctive points are identified in the image. A hash table is built, where the keys are ordered pairs from the distinctive points and the value is a collection of coordinates of the remaining points measured in a coordinates system given by the pair of key points. This is done for any two points from the object. To recognize an object in an image, the same is done for two arbitrarily chosen distinctive points. If the object is “known”, there will be a slot in the hash table that has a very similar collection of points relative to their key points, and thus the object can be predicted. Geometric hashing is robust to object rotation, translation, small variations in object shape and to partially occluded objects.

When transferring this concept to seed finding for pairwise sequence alignments, matters even get easier. The “objects” we try to identify are orthologous genes. Exons of orthologous genes are much better conserved than typical noncoding sequences, thus we expect many matches there, while there should be only few matches in the less conserved introns and intergenic region. The seeds are our distinctive points. The only transformation we require is horizontal shift. In fact, there is no need to limit geometric hashing to two genomes. It is easily extensible to process seed candidates of more than two genomes. We here first show the special case of two-dimensional seed candidates that was used to compare geometric hashing to the other methods. Later, we generalize the approach to higher dimensions.

**Fig. 1:**
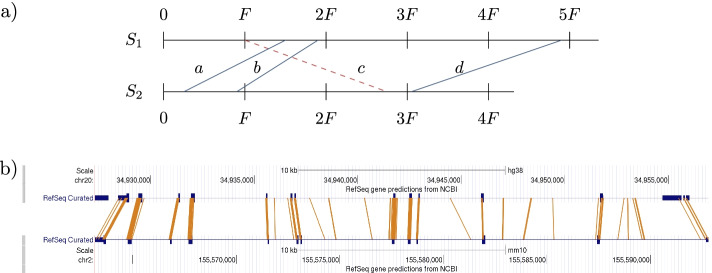
Geometric hashing. **a** Idea of geometric hashing. Seeds *a*, *b* and *d* map geometrically to the same tile $(S_1,S_2, 1)=g(a)=g(b)=g(c)$ and support each other even though they are distant and there are indels between them if they specify homologous site pairs. Seed candidate *c* maps geometrically to tile $g(c)=(S_1,S_2,-2)$ whose significance falls below the threshold and is not reported as no other seeds map geometrically to the same tile. **b** Seeds from human and mouse gene *glutathione synthetase* (*GSS*, Ensembl IDs ENSG00000100983 and ENSMUSG00000027610, respectively). Conserved exons (thick blue bars) are hit by many seed matches (orange lines). All seeds from the $\approx \,30\,kpb$ gene range were collected in a single tile, despite differing intron lengths between corresponding exons. Edited screenshots from UCSC Genome Browser [47, 48]

Two seed candidates ($S_1,i,S_2,j$) and ($S_1,i',S_2,j'$) from different exons from the same two orthologous genes may be thousands or even ten thousands of basepairs apart in the genome. Yet, the relative distances $i-j$ and $i'-j'$ are often similar. Using this relative distance, we are able to collect seeds from even very distant exons. As the interjacent introns often have undergone length changes through insertions or deletions, we round the relative distances to multiples of $F=10{,}000$ bp (‘quantization’). *F* is a parameter and its value is here set for vertebrate-sized genomes. This way, the relative distances of seeds become robust to varying intron lengths up to a certain degree. See Fig. 1b where this is illustrated. It displays two sequences $S_1, S_2$ from human (top) and mouse (bottom), which turn out to be the orthologous glutathione synthetase (GSS) genes. The thick blue bars are annotated exons, which the algorithm is unaware of. The orange lines are seeds found by our geometric hashing approach. Some introns quite visibly differ in length, yet the seeds all were collected at one place we call a *tile*. A more formal description of the geometric hashing approach follows.

Let *S* be a set of candidate seeds, e.g. from either method of M1-M4. Let *F* be a *tile size* (we use $F=10,000$ for vertebrate genomes) and define a *geometric map*

1 $$\begin{aligned} g(S_1,i,S_2,j) := (S_1,S_2,\left\lfloor (i-j)/F\right\rfloor ) \end{aligned}$$

that maps a candidate seed to the pair of sequence identifiers and a difference tile. Here, $\lfloor \; \rfloor$ means rounding down to the next integer. Let $T:=g(S)$ be the image set of the geometric map. We call the elements of *T* *tiles*. For a tile $t\in T$, the set $g^{-1}(t)$ contains seeds of the same sequence pair that have similar differences $i-j$:

$$\begin{aligned} g(S_1,i,S_2,j) = g(S_1,i',S_2,j') \Rightarrow |(i-j) - (i'-j')| = |(i-i') - (j-j')| < F \end{aligned}$$

See Fig. 1a for an illustration of the idea. One can think of a tile as a diagonal stripe of width *F* in the pairwise alignment space.

**Fig. 2:**
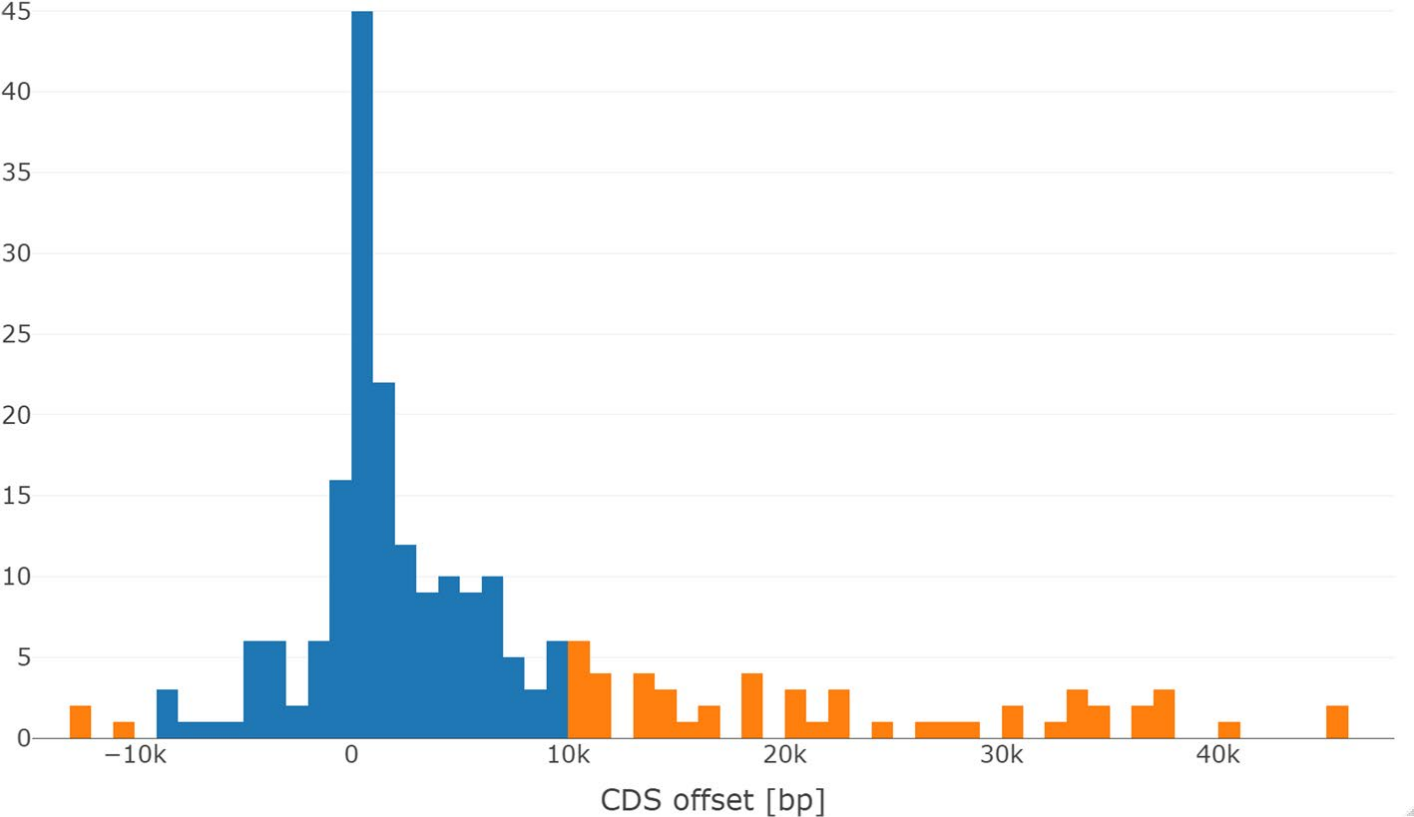
Distribution of maximal exon offsets $\Omega$ in the test set of human-mouse orthologs. Exon offsets measure the cumulative effect that indels have on the relative positions of exons. For most genes, maximal exon offsets are in the range $[-10{,}000\,\hbox {bp},10{,}000\,\hbox {bp}]$ (blue). The offsets whose absolute value is beyond $F=10{,}000$ are marked in orange. The distribution leans to the right, apparently because there are more transposable elements inserted in intronic regions in the human lineage. Outliers are not shown. See main text for the definition of $\Omega$

The choice of a tile size in the order of 10,000 bp was based on an analysis of our dataset (Fig. 2). The choice constitutes a tradeoff. Ideally, the tile size would be big enough so that even genes with greatly differing intron lengths are not ‘broken‘ into neighbouring tiles. On the other hand, very large tile sizes may lead to spurious tiles that by chance contain many uninformative seed candidates.

To study the effect of indels on the relative positions of exons of orthologous human-mouse pairs of genes, we filtered the human and mouse gene pairs from our data such that all pairs have the same respective number of CDS and assumed that identically numbered CDS are orthologous. We then calculated the maximum offset between ‘orthologous‘ CDS: Say there are *n* CDS in a particular gene and denote the start of the *i*-th CDS in human with $a_i^1$ and in mouse with $a_i^2$. The maximum offset is then defined as $\Omega :=\max _{i=1}^n |(a^1_i\,-\,a^1_1)\,-\,(a^2_i\,-\,a^2_1)|$. When looking at the distribution of maximum offsets (Fig. 2), we see that with a tilesize of 10,000 bp we can catch approximately $75\%$ of genes in a single tile, while results show a reasonable $\widehat{\text {FP}}$. Organisms with smaller average intron lengths can be expected to also have smaller offsets as well, so that a smaller parameter *F* may be better.

After performing geometric hashing, the encountered tiles are scored and all seed candidates from a tile *t* are reported as seeds if the score of *t* matches or exceeds a threshold $\tau$. If the threshold is not reached, none of the seed candidates of the tile are output. The tile scoring algorithm works as follows. Let $|S_1|$ and $|S_2|$ denote the lengths of sequences $S_1$ and $S_2$, respectively. We divide each tile into a fixed number of $b$ sub-tiles of equal width $F$/*b* and the sub-tiles further into chunks of length $h$.

Each seed candidate $(S_1,i,S_2,j) \in g^{-1}(t)$ in a tile *t* is assigned to a chunk $\lfloor (i+j) / h \rfloor$ in a sub-tile $\lfloor d b / F \rfloor \in \{0,\ldots , b-1\}$, where $d = i - j - t^*$ is the seed candidate’s diagonal inside *t* and $t^* := F \cdot \lfloor (i-j)/F\rfloor$ is the first diagonal of tile *t*. We found that with our data, $b = 50$ sub-tiles and chunk length $h = 400$ work well. We denote with $n_{r, s}$ the number of seed candidates in sub-tile $r \in \{0,\ldots , b-1\}$ and chunk $s \in \{0,\ldots , s_{max}\}$ of *t*, with $s_{max} = \lfloor (i^*+j^*) / h \rfloor$ such that $s_{max}$ is maximal given $g(S_1,i^*,S_2,j^*) = t$ and $i^* \in [1,\ldots , |S_1|]$ and $j^* \in [1,\ldots , |S_2|]$. We then calculate the *p*-norm for the score (Equation (2)). This gives a relatively higher score to tiles whose seed candidates cluster on few chunks and less so for evenly scattered seed candidates.

The former we expect in truly orthologous sequences where single orthologous exons share many seeds on similar diagonals (see Fig. 1b), the latter we expect from random or unrelated sequences.

With longer sequences $S_1,S_2$, we expect more spurious seed candidates appearing by chance and normalize the score to account for this noise. We compute the expected number $\lambda$ of seed candidates in a chunk when all seed candidates were evenly scattered across the genomes as $\lambda = A L / (n_1n_2)$ with *A* denoting the “area” of a chunk, i.e. $A = h F / b$, *L* the total number of observed seed candidates in all tiles and $n_1, n_2$ denoting the sum of sequence lengths (both genomic and artificial) in human or mouse, respectively. We use $\lambda \cdot b (s_{max}+1)$ as a normalization term, i.e. $\lambda$ times the number of chunks in tile *t*. The score for tile *t* is then

2 $$\begin{aligned} \text {Score}(t) := \frac{1}{\lambda b (s_{max}+1)} \root p \of {\sum _{r=0}^{b-1}\sum _{s=0}^{s_{max}}n_{r,s}^p}, \end{aligned}$$

where *p* is a parameter to adjust the impact of chunks that have more seed candidates than expected on the score. We used $p = 6$, such that the score is dominated by the chunks of sub-tiles with the most seed candidates.

#### Two-step geometric hashing

Separating the alignment space into tiles yields another possibility to increase specificity. As stated above, exons from one gene often all lie in the same or few neighbouring tiles, given a sufficient tile size *F*. To find seeds in these exons, it is sufficient to only search inside these tiles, thus finding these tiles before the actual seed finding would result in having fewer spurious or FP seed candidates and in a great speedup.

This approach uses two different seed pattern weights $k$ and $k'$ with $k < k'$. Using a high weight $k'$, seed candidates can be counted for each tile in a first run. A high weight seed mask is not very sensitive but highly specific. By using a simple link count threshold $\tau '$, we can select a set $T'$ of tiles that have $\tau '$ or more seed candidates. To catch more exons, we also include all the neighbouring tiles $t+1, t-1 \forall t \in T'$. Then, in a normal geometric hashing run with a (set of) sensitive low weight seed mask(s) with weight *k* as described above, we only consider seed candidates $(S_1,i,S_2,j) \in g^{-1}(t)$ if $t \in T'$. Thus, if we catch the right tiles in the first run, we can find all exons inside these tiles in the second run.

#### Multi-genome seed finding

The geometric hashing approach can be generalized to find seed candidates when comparing more than two genomes. This issue can arise when a non-progressive multiple genome alignment method is developed. Let $$s\ge 2$$ be the number of homologous input sequences. A (multiple alignment) seed candidate is then a tuple $$(S_1,i_1, S_2,i_2, ..., S_s, i_s)$$. For the exact contiguous matching method (M1) such a seed candidate means that there was an identical substring $$S_1[a_1 .. a_1+k) = S_2[a_2 .. a_2+k) = ... = S_s[a_s .. a_s+k)$$ in each input sequence, where $$i_j = a_j + \lceil k/2\rceil$$ for $$i \in 1..s$$. This applies analogously for the spaced seed methods (M2, M3). The generalized geometric mapping function is

$$\begin{aligned} g(S_1,i_1, S_2,i_2, ..., S_s, i_s) := (S_1, S_2, ..., S_s, \lfloor (i_1-i_2)/F\rfloor , ..., \lfloor (i_1-i_s)/F\rfloor ). \end{aligned}$$

Here, $S_1$ is arbitrarily chosen as a *reference* sequence in order to resolve the overparametrization of the relative positions by *s* absolute positions. The matching positions in the remaining sequences are determined w.r.t. this reference sequence. The elements of the image set of mapping *g* are again called ’tiles’ and can now also represent a higher-dimensional quantization. Again, we propose to subdivide each tile into *b* roughly equally sized sub-tiles $0,\ldots , b-1$ and each sub-tile into chunks of length *h* in order to reward the case where multi-dimensional seeds that map to the same tile have even more similar relative position offsets than required by merely mapping to *t*. Let $n_{r,s}$ be the number of seed candidatates that map to the *s*-th chunk in the *r*-th sub-tile of *t*. The scoring formula (2) can then be applied also in this more general case as the fundamental reasoning did not change.

Geometric hashing is particularly suited for a generalization of the seed concept to multiple alignment, as it does not require an explicit comparison between pairs of seeds. This is a property that distinguishes geometric hashing from the seed grouping approach of YASS, for example. To our knowledge, the use of multi-dimensional seeds for multiple genome alignment has so far not yet been described.

### Geometric hashing algorithm

We here outline the geometric hashing algorithm (M5) with pseudocode and provide a runtime analysis. The algorithm for M4 (Neighbouring Matches) is detailed in the Additional file 1.

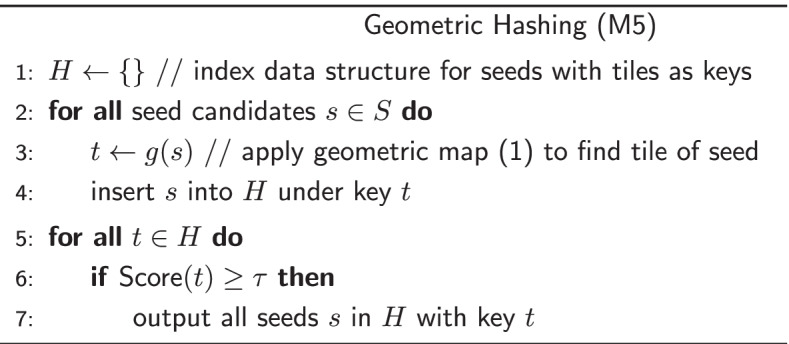



Our implementation in C++ uses a hash table for the data structure *H*. Inserting and querying elements from such a data structure can be done in *O*(1) expected time. The key of the hash table is a tile, and the value is an unordered set of seed candidates that fit into the tile. Inserting a seed candidate into an unordered set also takes *O*(1) time. Computing the score in line 6 is linear in the number of seed candidates in the tile.

## Results

**Fig. 3:**
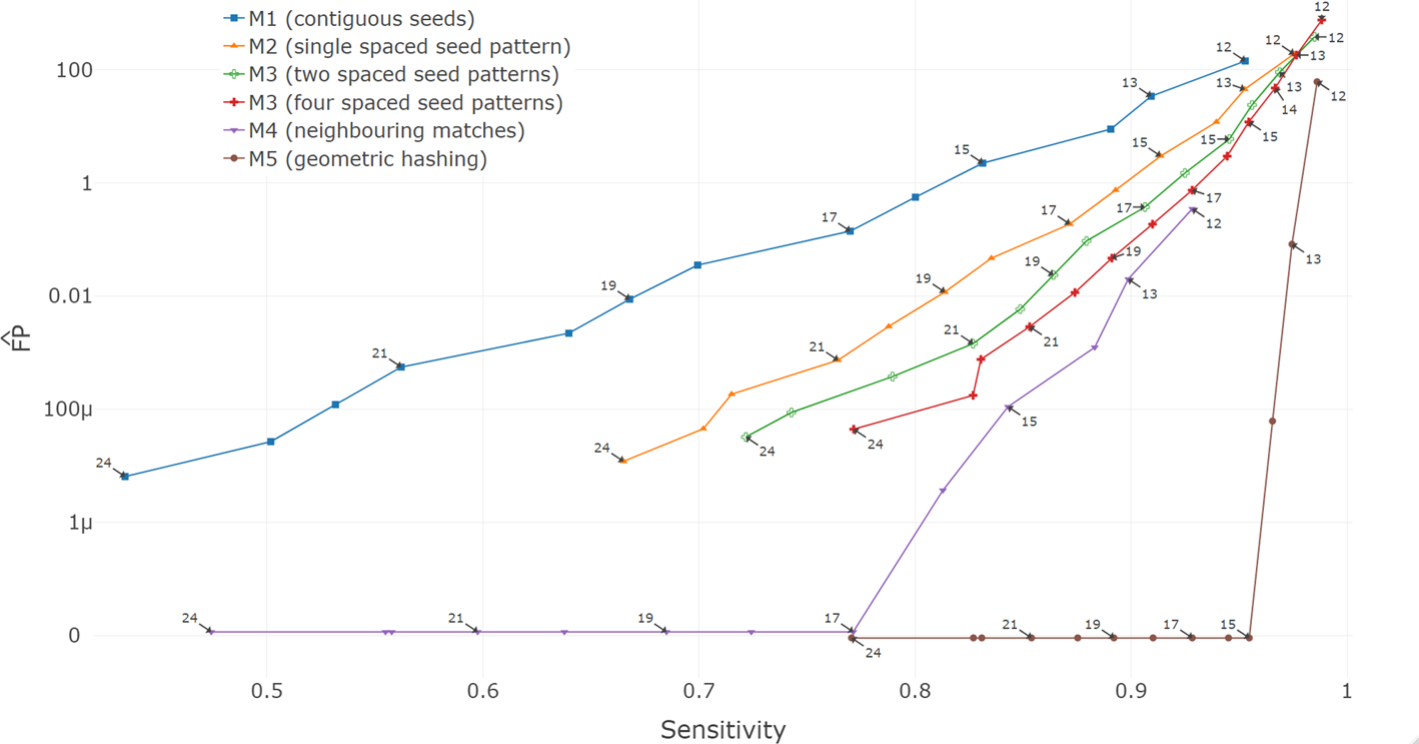
Comparison of the different methods. The *y*-axis shows on a logarithmic scale the total number of false positive seeds that are scaled to be estimates of the total number of false positive seeds per base in the genome if two complete human-sized genomes were compared ($\mu$ means $10^{-6}$). The *x*-axis is the percentage of coding exons that are supported by seeds. Each data point represents a run of the respective method with a certain weight *k* (point labels), ranging from 12 (top right points) to 24 (bottom left). Note that data points at 0 $\widehat{\text {FP}}$ are slightly shifted for better visibility. The filtering to consider at most 10 seed candidates per *k*-mer was relaxed to 100 for all weight 12 runs

Figure 3 shows the extrapolated false positive fractions and sensitivities for all five methods, when the weight *k* and the number of spaced seed patterns (for M3) is varied. M5 was run as described in “M5: geometric hashing” section, without using the two-step approach. A decreasing *k* leads to larger sensitivities but the number of false positives grows exponentially. With a weight $k=12$ all methods except M4 are expected to produce 100 or more times as many seeds or intermediate seed candidates (M5) as there are bases in one genome, when seeds in two human-sized genomes are searched. For such tasks, a further decrease of *k* could quickly render seed finding or seed extensions computationally infeasible or have prohibitive memory usage.

On our test data set of unrelated sequences, geometric hashing completely removed all false positive seeds for all $k\ge 15$, while maintaining a very high accuracy (bottom right data point in Fig. 3). For methods that predicted 0 false positives in these random sequences (M4 for $k\ge 17$ and M5 for $k\ge 15$), the confidence interval for $\widehat{\text {FP}}$ is $[0, 4.2\cdot 10^{-6}]$ (confidence level $1-\alpha = 99\%$, assumption that #FP is Poisson-distributed).

Consider the comparison of the red and the brown points labeled $k=15$ in Fig. 3. Here, M5 (brown) received the seeds output from M3 (red) as input and provided an additional filter. With it, geometric hashing reduced the number of false positives produced by a set of four spaced seed patterns (M3) of weight $k=15$ from 12 false seeds per genome position (#FP = 13,035,210) to arguably less than $6.2\cdot 10^{-6}$ false seeds per genome position (#FP = 0), i.e. by a factor of about $2\cdot 10^6$. At the same time, the sensitivity did not decrease.

When using a smaller *k*, seed finding with geometric hashing can simultaneously be more sensitive and much more specific than sets of spaced seeds. To see this, compare geometric hashing (M5) for $k=14$ with M3 (four patterns) for $k=15$. With this configuration, M5 has 3.5% false negatives and 67 FP while M3 has 4.6% false negatives and 13,035,210 FP (see Fig. 3 and Additional file 1). Stated differently, M5 reduces the false negatives by $0.9\% / 4.6\% \approx 19.5\%$ and the false positives by a factor $\sim 2\cdot 10^5$.

**Table 1:** Runtime and memory requirements of comparable runs of M3 (multiple spaced seed patterns), M4 (neighbouring matches) and M5 (geometric hashing)

Method	Weight	Patterns	Sensitivity	$\widehat{\text {FP}}$	Additional runtime (s)
M3	15	4	0.954	12	–
M4	15	4	0.843	$11\times 10^{-5}$	86
M5	15	4	0.954	0	300

M4 (neighbouring matches) is able to achieve zero false positives as well, however at lower sensitivity. Among the baseline methods M1-M3, contiguous seeds have the worst trade-off between sensitivity and $\widehat{\text {FP}}$. Using one spaced seed pattern greatly improves this trade-off, which is even better when using two or four spaced seed patterns. M4 and M5 were run with four spaced seed patterns and are thus directly comparable to the performance of M3 with four patterns. We compared the runtime of M3, M4 and M5 when all were run with the same spaced seed patterns in Table 1. M4 and M5 both need only marginally more time to improve the specificity. However, M4 reduces the sensitivity stronger than M5 compared to M3.

### Metaparameter optimization

**Fig. 4:**
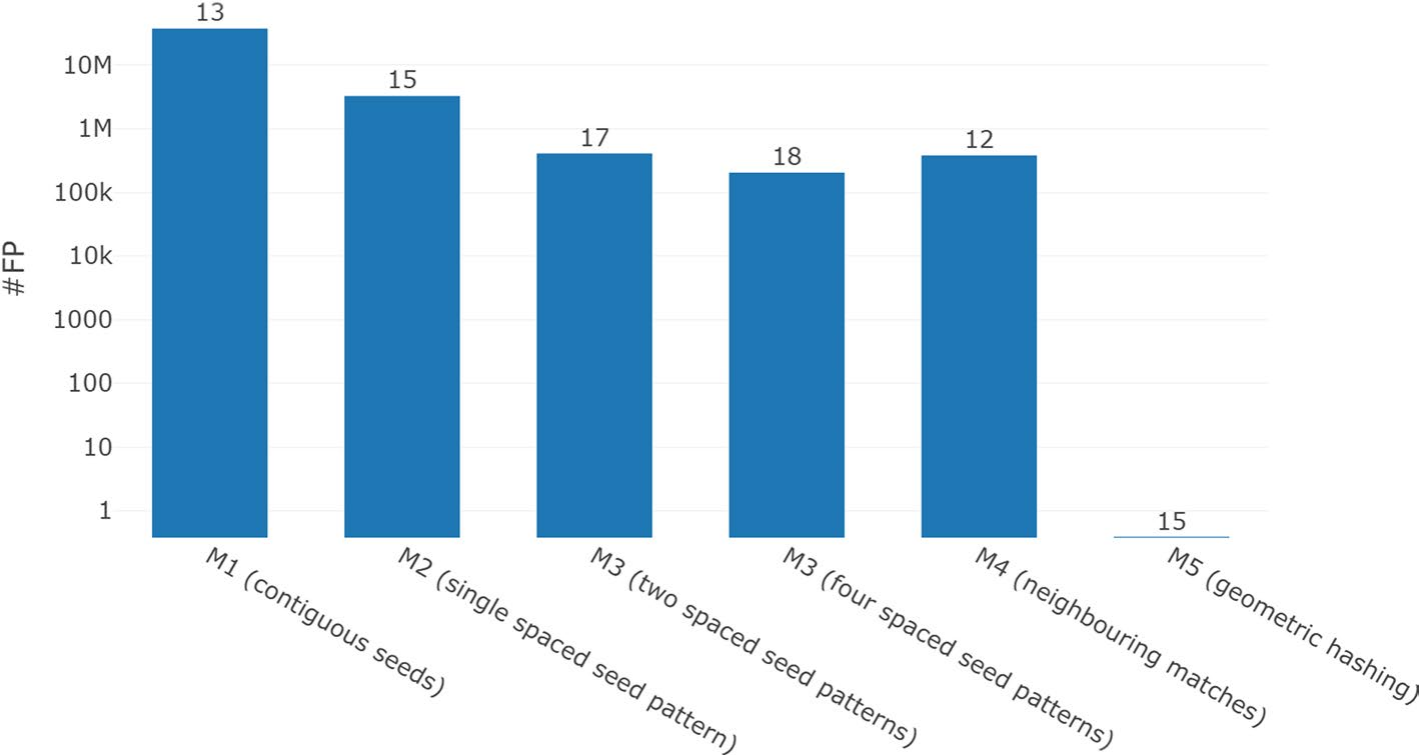
Comparison of minimal achievable FP count when sensitivity is required to be at least 0.9. Geometric hashing is the only method that does not report any false positives in our dataset at this sensitivity threshold. The numbers on top of the bars denote the weight of the seed or spaced seed pattern(s). On the *y*-axis k and M abbreviate $10^3$ and $10^6$, respectively

**Table 2:** Runtime and memory requirements of the methods when run with best weight as determined by Fig. 4

Method	Weight	Patterns	Sensitivity	$\widehat{\text {FP}}$	Runtime (min)	Memory (GB)
M1	13	1	0.909	34.3	32	38
M2	15	1	0.914	2.99	17	26
M3	17	2	0.906	0.375	38	64
M3	18	4	0.909	0.0116	87	156
M4	12	4	0.928	0.349	194	80
M5	15	4	0.955	0	75	137

The weights were chosen such that each method had the lowest possible $\widehat{\text {FP}}$ but a sensitivity of at least 0.9. Note that the runtime and memory for M4 cannot be directly compared to the other methods as we needed to split the data into batches and do multiple sequential runs because of memory limitations

The key parameter for seed finding is the weight *k* of the seeds. We determined optimal weights minimizing $\widehat{\text {FP}}$ while requiring that the sensitivity is *at least* 0.9. Figure 4 shows the FP count for each method at the optimal weight (numbers over the bars). In Table 2 we compare the performance of the respective runs. Using multiple spaced seeds (M3) alone heavily increases runtime and memory requirement, which can be reduced with smaller weights. Even though geometric hashing is neither the fastest nor requires the least memory, it yields the highest sensitivity as it produced no FPs. Note that we also did not focus on optimizing our code to get the best computational performance possible. We performed grid searches on the remaining metaparameters for the respective methods. Neighbouring matches (M4) was run with a neighbour count threshold $\tau = 2$ and a search area $D = 1000$. Geometric hashing was run with a tile size $F = 10,000$, $b = 50$ sub-tiles, chunk length $h = 400$ and $p = 6$. The tile score thresholds $\tau$ were optimized for the different weights independently (see Additional file 1).

### Comparison with competing tools

To evaluate the performance of *geometric hashing* in comparison to other state of the art seed filtering programs, we ran the YASS alignment software on our dataset. YASS uses the “group criterion” [27] for seed filtering, of which our M4 is a simplified version. They also use special “transition-constrained” seeds. We evaluated the results in a very similar manner to our seed finding methods. Each human exon that was covered by an alignment was considered “supported” and, as before, sensitivity = number of supported human CDS/number of human CDS. For the false positives we counted all the alignments between randomly generated sequences. *Geometric hashing* results were evaluated as before. Using the two-step approach (“Two-step geometric hashing” section), *geometric hashing* is able to outperform YASS on our dataset in terms of sensitivity, FP count and runtime, see Table 3. We used an optimal single spaced seed mask of weight $k' = 19$ in the first step and four optimal spaced seed masks of weight $k = 8$ in the second step. The spaced seed masks were again computed using SpEED [17, 40]. The link count threshold $\tau '$ was 3, the tile scoring threshold $\tau$ was also 3. All other parameters were set as before. We used default YASS parameters after confirming in a grid search that they perform best.

**Table 3:** Comparison of YASS [27] and *geometric hashing* using the two-step approach (“Two-step geometric hashing” section) on the same dataset as used in Fig. 3. Sensitivity is the fraction of human conding exons covered by an alignment for YASS or by a seed for geometric hashing

Method	Sensitivity	FP	Runtime (min)	Memory
YASS	0.9931	7	107	590 MB
Geometric hashing	0.9979	0	49	28 GB

FP denotes the number of alignments between randomly generated sequences in YASS and the number of such seeds for geometric hashing

### Very long input sequences

In our global seed-finding strategy, a decision to include a seed can potentially be influenced by very far away sequence positions. To test this long-range influence, we performed an experiment with the complete chromosomes 20 and 2 of human and mouse, respectively, as input. These chromosomes share 151 ortholog genes from our data set. We used these genes to assess the sensitivity as described in “Methods” section. Here, the sensitivity analysis is restricted to the subset of these 151 genes and the second seed position is required to be in the range of the respective orthologous mouse gene. The number of false positive seeds is again estimated on two random sequences of lengths of the respective chromosomes, 64Mb and 182Mb. For direct comparison, we ran geometric hashing also on the subset of 151 gene pairs. We made this experiment with a weight of $k=15$ and both runs were performed with the same parameters. In both runs, the number of false positives (FP) was zero. The sensitivities when inputting two whole chromosomes or only gene pairs were 0.978 and 0.954, respectively. This shows that in principle our method also works on bigger inputs like whole chromosomes. However, we observed a longer runtime (98 min vs. 11 min) and higher memory consumption (153 GB vs. 26 GB) in the whole-chromosome run vs. the gene pair run as the input is much larger. The sensitivity for the whole chromosome run is somewhat higher. We suspect this is because geometric hashing can now benefit from synteny effects: A human CDS that is missed when comparing two gene regions only, may be in a tile that reaches the tile scoring threshold $\tau$ when complete chromosomes are input that include nearby syntenic gene pairs.

## Discussion

Our experiments show that geometric hashing is the most accurate method on our test data. Geometric hashing can reduce the number of false positives by about 6 orders of magnitude over a common strategy in seed finding, using sets of (four) spaced seed patterns. The later method itself constitutes an improvement of 3–4 orders of magnitude over the naive method of using contiguous *k*-mer matches as alignment seeds on protein-coding genes.

Geometric hashing can be used as a filter on any other seed finding method and may potentially be ’inserted’ into the internal pipeline of existing alignment programs between seed finding and seed extension. A large speed-up may be possible in such aligners if significant parts of the runtime are spent downstream of seed finding. Note that seed extension, followed by scoring and filtering, is also an algorithmic step to achieve a higher specificity. However, it requires running an algorithm for local alignment scoring over a variable-length region. In contrast, geometric hashing only requires a constant number of simple integer arithmetic functions (in fact two: one for mapping, one for scoring) and does not require any sequence comparisons. A seed extension phase would still follow but needs to be executed on a much smaller set of seeds.

Moreover, a higher sensitivity can be achieved by lowering *k*. Due to the effective filtering of geometric hashing, this need not come at the computational cost of a large number of false positives. The seed finding with geometric hashing can then simultaneously be somewhat more sensitive and much more specific than sets of spaced seeds. Even small improvements in sensitivity may result in finding orthologies with lower sequence similarity [39].

With our results we confirmed the well established advantage of spaced seeds (M2, M3) over contiguous seeds (M1) and that additional filtering steps (M4, M5) can improve seed performance even further. The focus of this work was to show that considering even distant exons in filtering, as done in geometric hashing, works and is superior to only considering local neighbourhoods of seeds candidates as done in M4. Due to its ability to filter out FP efficiently, we expect a large runtime improvement from geometric hashing when applied in pairwise whole-genome alignments, as much fewer wrong seeds have to be considered in the more time consuming seed extension phase. Since geometric hashing is also easily generalizable to process seeds from an arbitrary number of input sequences in a truly simultaneous fashion, we think it is a promising novel tool in multiple genome alignment tasks.

Geometric hashing aggregates non-local similarities and, accordingly, we tested it here on input sequences that span at least a whole vertebrate gene. It can be expected that the relative advantage is lost, when only short similarities are sought, e.g. from small genome rearrangements or horizontal transfer of DNA fragments. However, we think that the geometric hashing idea is not limited to genome alignments. It could be adapted for protein sequences as well and with some adjustments also for protein to mRNA alignments, or it could be used to speed up tBLASTX [3], which finds region pairs in genomes that are similar peptide sequences when translated. As mentioned earlier, the geometric hashing idea also generalizes well for higher dimensional seeds that occur when multiple genomes are compared simultaneously. Future work could focus on the necessary adaptations for this to work well.

The methods for seed filtering can be seen as proofs of concept with much potential for improvements as we did not aim to write a fully optimized software. For example, it has been reported that sorting matches by keys rather than hashing can improve runtime due to better data locality [25]. Such an implementation is compatible with our geometric hashing approach, both for the primary hashing of *k*-mers and for the secondary geometric hashing of tiles. Further, the overall memory footprint can be decreased when the genomes are processed in chunks rather than all at once.

## Conclusion

We presented a novel seed filtering approach, *geometric hashing*, that uses non-local neighbourhood information to find orthologous genes with high precision. It outperforms local neighbourhood filtering while only slightly affecting the sensitivity of unfiltered seeds. Geometric hashing is a simple yet powerful idea and generalizes well to other alignment problems and higher dimensional data and could be a strong tool in future (multiple) genome alignment approaches.

## Supplementary Information

**Additional file 1.** The supplementary material includes additional tables and figures.

## Data Availability

The code and test data is available at our Github repository [46].
