# Supplementary material for: Global, highly specific and fast filtering of alignment seeds
Source: BMC Bioinformatics. 2022 Jun 10;23:225. doi: 10.1186/s12859-022-04745-4 (PMC9188137; doi:10.1186/s12859-022-04745-4)
Supplement: Supplementary file 1 — Additional file 1. The supplementary material includes additional tables and figures. [file 12859_2022_4745_MOESM1_ESM.pdf]

Global, Highly Specific and Fast Filtering of Alignment Seeds Supplementary Materials

Matthis Ebel[1,2], Giovanna Migliorelli[1,2], and Mario Stanke[1,2]

[1]Institute for Mathematics and Computer Science, University of Greifswald, Walther-Rathenau-Str. 47, 17489, Greifswald, Germany
[2]Center for Functional Genomics of Microbes, University of Greifswald, Felix-Hausdorff-Str. 8, 17489, Greifswald, Germany

May 11, 2022

1 M4: Neighbouring Matches Algorithm

For this method, the seed candidates (S_1, i, S_2, j) are sorted. The primary sort criterion is the sequence pair (S_1, S_2), the secondary sort criterion is the diagonal $i - j$, the tertiary sort criterion is the position i. This sorting is achieved in $O(n \log(n))$ time where n is the number of seed candidates. In line 3 of below algorithm, GetNextDiagonal() returns a list of seeds that all lie on the same diagonal $i - j$, one diagonal at a time. This is linear in the length d of the respective diagonal. The body of the loop in line 4 consumes each seed exactly once and is in total executed n times. The check in line 5 can be implemented $O(1)$ amortized time.

Neighbouring matches filter algorithm

```
1: Sort(S) // sort set S of seed candidates
2: repeat
3:     S' = GetNextDiagonal(S)
4:     for all s ∈ S' do
5:         if numNeighbors(s, S') ≥ τ then
6:             report s
7: until all diagonals processed
```

2 Raw Accuracy Data

Tables [1], [2], [3], [4] and [5] list the values plottet for each method in Figure 3 of the main text.

Supplementary Table 1: Details for M1 (contiguous seeds) for different weights

weight	patterns	sensitivity	$\widehat{FP}$	#FP
12	1	0.953	144	156570904
13	1	0.909	34.3	37283878
14	1	0.891	8.98	9775062
15	1	0.831	2.25	2445475
16	1	0.800	0.561	610832
17	1	0.770	0.141	153159
18	1	0.699	0.0353	38446
19	1	0.668	0.00881	9591
20	1	0.640	0.00221	2400
21	1	0.562	0.000555	604
22	1	0.532	0.000120	131
23	1	0.502	0.0000267	29
24	1	0.434	0.00000643	7

Supplementary Table 2: Details for M2 (single spaced seed pattern) for different weights

weight	patterns	sensitivity	$\widehat{FP}$	#FP
12	1	0.975	192	208560935
13	1	0.953	45.6	49583001
14	1	0.940	12.0	13038891
15	1	0.914	3.00	3260194
16	1	0.893	0.749	815384
17	1	0.872	0.188	204164
18	1	0.835	0.0468	50909
19	1	0.814	0.0118	12812
20	1	0.788	0.00290	3159
21	1	0.764	0.000734	799
22	1	0.715	0.000184	200
23	1	0.702	0.0000450	49
24	1	0.665	0.0000119	13

Supplementary Table 3: Details for M3 (multiple spaced seed patterns) for different weights

weight	patterns	sensitivity	$\widehat{FP}$	#FP
12	2	0.985	383	416913389
13	2	0.969	91.4	99410073
14	2	0.956	24.0	26068083
15	2	0.945	5.99	6516965
16	2	0.925	1.50	1630306
17	2	0.906	0.375	407886
18	2	0.879	0.0943	102640
19	2	0.864	0.0235	25598
20	2	0.849	0.00587	6388
21	2	0.827	0.00144	1567
22	2	0.790	0.000377	410
23	2	0.743	0.0000873	95
24	2	0.722	0.0000322	35
12	4	0.988	764	831774401
13	4	0.977	183	199134962
14	4	0.967	47.9	52074145
15	4	0.954	12.0	13035210
16	4	0.944	2.99	3258172
17	4	0.928	0.750	815647
18	4	0.910	0.188	204039
19	4	0.891	0.0470	51156
20	4	0.874	0.0116	12594
21	4	0.853	0.00287	3126
22	4	0.830	0.000760	827
23	4	0.827	0.000176	191
24	4	0.772	0.0000441	48

Supplementary Table 4: Details for M4 (neighbouring matches) for different weights

weight	patterns	sensitivity	$\widehat{FP}$	#FP
12	4	0.928	0.349	380231
13	4	0.899	0.0200	21742
14	4	0.883	0.00124	1350
15	4	0.843	0.000110	120
16	4	0.813	0.00000368	4
17	4	0.771	0	0
18	4	0.724	0	0
19	4	0.685	0	0
20	4	0.638	0	0
21	4	0.598	0	0
22	4	0.558	0	0
23	4	0.555	0	0
24	4	0.474	0	0

Supplementary Table 5: Details for M5 (geometric hashing) for different weights

weight	patterns	sensitivity	$\widehat{\text{FP}}$	#FP	τ
12	4	0.986	61.8	67283629	41
13	4	0.974	0.0826	89902	206
14	4	0.965	0.0000616	67	1552
15	4	0.955	0	0	6156
16	4	0.945	0	0	19366
17	4	0.928	0	0	52575
18	4	0.910	0	0	101209
19	4	0.892	0	0	130324
20	4	0.875	0	0	115719
21	4	0.854	0	0	150655
22	4	0.831	0	0	134879
23	4	0.827	0	0	155271
24	4	0.771	0	0	149653
